# The association of body composition and fat distribution with dysmobility syndrome in community-dwelling older adults: Bushehr Elderly Health (BEH) program

**DOI:** 10.1186/s12891-023-06934-5

**Published:** 2023-10-12

**Authors:** Mohammad Mehdi Khaleghi, Hadi Emamat, Maryam Marzban, Akram Farhadi, Ali Jamshidi, Negin Ghasemi, Azar Falahatzadeh, Zahrasadat Jalaliyan, Hasan Malekizadeh, Iraj Nabipour, Bagher Larijani

**Affiliations:** 1 Student Research Committee, Bushehr University of Medical Sciences, Bushehr, Iran; 2 The Persian Gulf Tropical Medicine Research Center, The Persian Gulf Biomedical Sciences Research Institute, Bushehr University of Medical Sciences, Bushehr, Iran; 3 Department of Nutritional Sciences, School of Health, Bushehr University of Medical Science, Bushehr, Iran; 4 Statistical Genetics Lab, QIMR Berghofer Medical Research Institute, Brisbane, QLD Australia; 5 School of Medicine, Bushehr University of Medical Sciences, Bushehr, Iran; 6 The Persian Gulf Marine Biotechnology Research Center, the Persian Gulf Biomedical Sciences Research Institute, Bushehr University of Medical Sciences, Bushehr, Iran; 7 Endocrinology and Metabolism Research Center, Endocrinology and Metabolism Clinical Sciences Institute, Tehran University of Medical Sciences, Tehran, Iran

**Keywords:** Musculoskeletal disorders, Functional decline, Fat distribution, Anthropometric index, Body composition index, Elderly

## Abstract

**Background and objective:**

Dysmobility Syndrome (DS) is characterized as an accumulation of clinical risk factors for functional disability, such as osteoporosis, sarcopenia, and obesity. Neurological disorders that affect the motor and sensory systems can also contribute to the condition, resulting in gait and muscle strength disturbances, as well as a history of falls and fractures. The study aimed to determine the association between fat distribution in different body areas and the odds of older adults developing DS, as there is still uncertainty about the accumulation of fat in which area is most closely linked to the condition.

**Methods:**

This cross-sectional study was conducted according to the data from the second phase of the Bushehr Elderly Health Cohort (BEH). Dysmobility Syndrome was defined based on the co-occurrence of at least three outcomes of its criteria. Body composition was measured using dual-energy X-ray absorptiometry (DXA) and anthropometric studies. For evaluating the relationship, multivariate logistic regression and adjusted univariate linear regression were used.

**Results:**

Of 2,359 who were recruited in the study, 1,277 participants (54.13%) had DS. According to the final logistic regression model in the limb region, FM and FM to FFM ratios were significantly associated with DS [OR (95%CI) = 1.04 (1.02 to 1.05), and 3.42 (1.95 to 5.99), respectively]. Also, In the trunk region, the FM and FM to FFM ratio were significantly related to the odds of DS, although this relationship was weaker than in the limbs region [OR (95%CI) = 1.02 (1.00 to 1.03), and 2.45 (1.36 to 4.39), respectively].

**Conclusion:**

Our findings indicate that a higher regional and whole-body amount of fat mass rather than fat-free mass is closely linked to an increased risk of DS, particularly in the elderly population. Notably, higher fat mass in the limbs (especially in the legs) is associated with greater odds of DS, while a higher android-to-gynoid fat mass ratio is associated with lower DS risk. Screening fat mass distribution in older individuals can be a valuable strategy for promptly diagnosing DS, implementing interventions to prevent disabilities, and improving their quality of life.

## Introduction

The Obesity epidemic is a serious public health issue for different societies, especially for the older people around the world [1–3], which can cause various complications; such as high risk of falling [4], musculoskeletal disorders and mobility disabilities [5]. Dysmobility Syndrome (DS) is a disability that can affect older adults and obese individuals. It is a new term introduced by Binkley and colleagues and encompasses various clinical risk factors that can lead to functional disability and adverse health outcomes in older individuals. The syndrome is characterized by six factors, including osteoporosis, occurrences of falls in the past year, obesity, low lean mass, slow gait speed, and low grip strength. DS is diagnosed with three or more factors present, regardless of specific prerequisites according to Binkley classification [6]. This definition has been used several times in subsequent studies [7, 8]. DS is defined with a score-based approach, although measuring skeletal muscles is challenging since low muscle mass individuals identified with weight-adjusted muscle index might tend to be overweight and obese; and height-adjusted muscle index-identified low muscle mass tend to lean [9].

The prevalence of DS varies according to the measurements used to define the syndrome and the selected population [6]. Among 6070 Korean women with an average age of 74.1 years, only 43 (0.7%) participants were suffering from DS [10]. On the other hand in an elderly cohort study in Taiwan, the prevalence rate was 3.9–10.1% [9], and in a systematic review study conducted in the same year, the prevalence rate was reported as between 22–34% [11]. Also, in a study conducted on Mexican postmenopausal women, the prevalence rate was 74%, which to the best of our knowledge is the highest prevalence rate reported among current studies [12].

Although there are no accurate statistics on the status of DS in Iran, but due to the aging of the population, the prevalence of related functional disabilities, including sarcopenia, osteoporosis, and obesity, is increasing in Iran [13].

The risk factors for DS are female sex and older age [6, 14, 15], fragility fracture [16], fractures that have happened in the past [14, 16], sarcopenia [12, 17], osteopenia and osteoporosis [12, 18], falls [11, 12], having chronic diseases [16], metabolic syndrome components [18], history of arthritis [18], less physical activity [16, 19], alcohol consumption [16], and obesity [20].

Studies have found that if older people suffer from obesity, the risk of falling and the prevalence of immobility in them increases [21]; These are risk factors for the occurrence of more dysmobility. On the other hand, we also know that obesity and the form of fat distribution in the body can be a risk factor for increasing the prevalence of sarcopenia [22], and osteoporosis [23]; which can increase the possibility of more fractures due to falls [21, 24]. A study showed that in sarcopenic obesity, which is defined as visceral obesity, a higher android to gynoid fat ratio (A/G ratio) is associated with the risk of vertebral fractures due to osteoporosis [25]. In another study conducted on healthy Thai women aged 40 to 90 years, higher android and gynoid obesity were associated with higher bone mineral density (BMD). In further investigations, it was seen that gynoid obesity has a stronger positive relationship with BMD instead of android obesity. This condition can be considered a protective effect for bone mass in postmenopausal Thai women [26]. In another study, A/G ratio was one of the most important factors in predicting the hip fracture pattern after falling in older patients [27]. So according to the existing definitions of DS and other information, it can be concluded that DS can be one of the complications of obesity.

Notably, although Fat Mass (FM) is a crucial component to the diagnosis of DS, there remains a lack of clarity regarding which specific area of the body's fat distribution is most closely linked to this condition. Therefore, the present study aimed to investigate the relationship between anthropometric indices, body composition, and, particularly, the accumulation of fat in different areas of the body (namely arms, legs, limbs, trunk, and whole body) with the likelihood of older adults having DS. To the best of our knowledge, there is no study that has examined the association between body composition and fat distribution with DS.

## Methods

### Research design and participants

This cross-sectional study was conducted as part of first phase (Stage II) of the Bushehr Elderly Health Program (BEH) project, a prospective and population-based cohort study in Bushehr, southern Iran. The full methodology of this project has been reported in detail previously [28, 29]. During the years of 2015–2016, Stage II of the first phase was conducted, involving a total of 2,426 participants. In order to be included in the study, individuals had to meet specific criteria, including providing informed written consent, being 60 years of age or older, maintaining residency in Bushehr for at least one year prior to the commencement of the research, and expressing a desire to remain in the city for at least two years following involvement in the investigation. If a participant was unable to provide consent, their legal guardian consented or they were excluded from the study. This study included a total of 2,359 participants who had a completed databank.

In a confidential manner, using a valid questionnaire [28, 29], the demographic status, general health, mental and functional health, lifestyle, and medical history were collected.

### Dysmobility syndrome

Initially, each participant's medical history was assessed by general practitioners. Then, according to existing definitions, dysmobility syndrome was defined as having at least three of the following criteria [6, 7]. Obesity/high fat mass: Total body fat %: > 40 for females; > 30 for males, Low lean mass: Appendicular lean mass ≤ 5.45 kg/m^2^ (females) or ≤ 7.26 kg/m^2^ (males), Osteoporosis: T-score of ≤  − 2.5 at lumbar spine, femoral neck, or total proximal femur, Low grip strength: Hand-held dynamometer: < 20 kg (female); < 30 kg (male), Slow gait speed: < 1.0 m/s (comfortable speed), Falls in the preceding year: Self-report of one or more falls over the past 12 months (Table 1) [6].

**Table 1:** Indicators and cut-off points to diagnose DS (at least three of them are required)^a^

Factor	Recommended cut point
Obesity/high fat mass	Total body % fat: > 40 for females; > 30 for males
Low lean mass	Appendicular lean mass ≤ 5.45 kg/m^2^ (females) or ≤ 7.26 kg/m^2^ (males)
Osteoporosis	T-score of ≤ − 2.5 at lumbar spine, femoral neck, or total proximal femur
Low grip strength	Hand-held dynamometer: < 20 kg (female); < 30 kg (male)
Slow gait speed	< 1.0 m/s (comfortable speed)
Falls in the preceding year	Self-report of one or more falls over the past 12 months

*DS* Dysmobility Syndrome

^a^Data derived from Binkley et al. study [6]

### Anthropometry and body composition

Through physical examination, information related to anthropometric measurements including height and weight, body mass index (BMI), neck circumference (NC), Waist circumferences (WC), Hip circumference (HC), and Waist to hip ratio (WHR) were collected.

Body composition assessment was performed by using dual-energy X-ray absorptiometry (DXA) (DXA, Discovery WI, Hologic, Bedford, VA, USA), with minimal exposure to radiation. Using Hologic DXA system, APEX software, analysis of raw scans of bone breakdown, muscle tissue, and fat tissue, for different regions of the whole body, android and gynoid area, trunk, legs, and arms, was performed. To ensure accurate DEXA measurements, individuals were required to avoid wearing clothing with metal objects and jewelry, undergo fasting before the scan, maintain hydration, share information about recent contrast material injections, and provide details about any surgeries or metal Implants. These conditions were essential for improving the precision of bone density and body composition assessments. Elsewhere, the details of the examinations and measurements are described [29].

Total body mass (BM) and fat mass (FM) represent the sum of BM and FM across all reported regions. Total body and regional fat-free mass (FFM) was calculated by subtracting FM from BM. Limbs FM and FFM were obtained from the sum of FM and FFM of the arms and legs.

### Other variables

Smoking was classified into three categories: current cigarette or hookah users, former cigarette or hookah users who quit, and those who never smoke or hookah. The classification of alcohol consumption was also done in four groups: regular, occasional, non-drinkers, and those who refused to report their status. Using medical records, chronic diseases were defined as having a history of the following diseases; hypertension (HTN, systolic or diastolic blood pressure respectively ≥ 140 mmHg and ≥ 90 mmHg or using anti-HTN medication), chronic kidney disease (CKD), liver disease, cardiovascular diseases, thyroid disease, diabetes (which is defined as FPG ≥ 126, HbA1C ≥ 6.5 mg/dL or taking anti-diabetic medication), rheumatoid arthritis and osteoarthritis. The prevalence of chronic diseases was also reported in the following three categories: No history of chronic disease, history of one chronic disease and history of two or more chronic diseases.

The level of physical activity was evaluated, using a 20-items questionnaire designed for the Iranian population [30, 31]. Each activity was measured in hours and minutes and reported as MET-min after being multiplied by its duration. The sum of all activities was used to calculate total physical activity (MET/24h) and the degree of physical activity was categorized into five groups: No activity: 0–1; sedentary: 1–1.39; low active: 1.4–1.59; active: 1.6–1.89; and highly active: 1.9–2.5) [30, 31].

Other variables were defined as follows: age (years), sex (female, male), marital status (single, married, divorced and widow) and income level was considered based on the report of the Social Security Organization and the government, in three income groups: low, medium and high, respectively.

### Ethical considerations

This study is approved by the Research Ethics Organization and the Research Committee of Bushehr University of Medical Sciences (IR.BPUMS.REC.1401.172) and the Endocrine and Metabolism Research Institute affiliated to Tehran University of Medical Sciences. (Ethical Code: IR.TUMS.EMRI.REC.1394.0036). All methods were carried out in accordance with the approved protocols and in accordance with the Helsinki Declaration.

### Statistical analysis

Categorical variables were presented as numbers and percentages whereas mean values and standard deviation (SD) were used for continuous variables. Data normality was checked using the Kolmogorov–Smirnov test. Differences between the two groups were evaluated using t-test and weighted Chi-square for continuous and categorical variables, respectively. Multivariable logistic regression analyses were performed to assess the association between DS and a variety of anthropometric and body composition indices. We selected relevant confounders based on an extensive literature search for which a significant clinical and pathophysiological association with desired outcome and/or exposures were first assessed by univariate regression models; then, statistically significant covariates, which have clinical implications were included in the multivariable logistic regression models. Covariates were adjusted as: model 1 = sex; model 2 = model 1 + age; model 3 = model 2 + number of chronic diseases, income and marital; model 4 = model 3 + smoking statues and physical activity. All analysis was done in Stata MP (version 17), and *p*-value < 0.05 was taken as statistically significant for all analyses.

## Results

Of 2,359 who were recruited in the study, 1,277 participants (54.13%) had DS. The mean age of all participants was 69.3 ± 6.3, and 51.5% were female. Participants with DS were mostly female (65.6% vs. 34.4%, *p*-value < 0.001) and older (71.0 ± 6.9 vs. 67.3 ± 4.8 years old, *p*-value < 0.001). There were no statistically significant differences between participants with and without DS in smoking, alcohol consumption, and prevalence of chronic diseases, whereas marital state and income level had statistically significant differences (*p*-value < 0.001). In married subjects, the prevalence of the DS was lower. People with high income level were also less affected by DS.

Among the anthropometric indices, Height, Weight, BMI, NC, WC, and HC, were statistically lower in participants with DS. It was also seen in DXA analysis that Lean Body Mass (LBM), Appendicular Skeletal Muscle (ASM), Lumbar and Hip bone marrow density were statistically lower in participants with DS. Furthermore, it was observed that the individuals with DS exhibit sedentary levels of physical activity (no activity and sedentary: 82.6% vs. 69.5%), display inferior physical performance (8.84 vs 9.97) and lower Gait Speed (0.71 vs 1.0) and Handgrip (17.64 vs. 27.62) (Table 2).

**Table 2:** Characteristics of the study participants according to the existence of DS (*n* = 2,359)

		Total population 2,359	Normal population 1,082(45.87%)	DS population 1,277 (54.13%)	*p*-value ^a^
Age		69.3 ± 6.33	67.26 ± 4.79	71.02 ± 6.94	< 0.0001
Sex, female n (%)		1,215 (51.50)	377 (34.84)	838 (65.62)	< 0.001
Smoking, n (%)	None	720 (30.52)	351 (32.44)	369 (28.90)	0.176
	Past-cigarette or Hookah	1,145 (48.54)	511 (47.23)	634 (49.65)	
	current-cigarette or Hookah	494 (20.94)	220 (20.33)	274 (21.49)	
Alcohol, n (%)	Not Response	3 (0.13)	2 (0.18)	1 (0.08)	0.050
	Never	2,329 (98.73)	1,061 (98.06)	1,268 (99.30)	
	Occasionally	20 (0.85)	15 (1.39)	5 (0.39)	
	Regular	7 (0.30)	4 (0.37)	3 (0.23)	
Marital, n (%)	Divorced	20 (0.85)	7 (0.65)	13 (1.02)	< 0.001
	Married	1819 (77.11)	948 (87.62)	871 (68.21)	
	Single	19 (0.81)	6 (0.55)	13 (1.02)	
	Widow	501 (21.24)	121 (11.18)	380 (29.76)	
Income, n (%)	Low	498 (21.11)	178 (16.45)	320 (25.06)	< 0.001
	Middle	1330 (56.38)	592 (54.71)	738 (57.79)	
	High	531 (22.51)	312 (28.84)	219 (17.15)	
Chronic disease	None	240 (10.17)	117 (10.81)	123 (9.63)	0.463
	one	315 (13.35)	150 (13.86)	165 (12.92)	
	Two or more	1,804 (76.47)	815 (75.32)	989 (77.45)	
**Anthropometric measurement**					
Height (cm)		158.81 ± 9.13	162.62 ± 8.35	155.59 ± 8.51	< 0.0001
Weight (kg)		68.95 ± 12.44	73.33 ± 11.37	65.23 ± 12.1	< 0.0001
BMI (kg/m2)		27.35 ± 4.65	27.81 ± 4.46	26.96 ± 4.77	< 0.0001
NC (cm)		36.95 ± 3.58	38.10 ± 3.42	35.98 ± 3.43	< 0.0001
WC (cm)		98.43 ± 11.71	99.46 ± 10.73	97.57 ± 12.41	0.0001
HC (cm)		102.28 ± 9.65	103.10 ± 8.83	101.58 ± 10.24	0.0001
WHR		0.96 ± 0.07	0.96 ± 0.06	0.96 ± 0.08	0.2172
**Dual-energy X-ray absorptiometry**					
LBM (kg)		41.93 ± 8.13	46.17 ± 7.50	38.34 ± 6.80	< 0.0001
ASM (kg)		15.89 ± 3.63	17.84 ± 3.40	14.25 ± 2.93	< 0.0001
Total fat mass (kg)		25.67 ± 8.01	25.62 ± 8.01	25.72 ± 8.02	0.765
Total fat percentage (%)		37.57 ± 8.12	35.24 ± 7.99	39.54 ± 7.69	< 0.0001
Lumbar bone marrow density		0.89 ± 0.18	0.97 ± 0.16	0.83 ± 0.17	< 0.0001
Hip bone marrow density		0.99 ± 0.19	1.06 ± 0.14	0.93 ± 0.21	< 0.0001
**Activity**					
Physical activity, n (%)	No activity	137 (5.81)	20 (1.85)	117 (9.16)	< 0.001
	Sedentary	1670 (70.79)	732 (67.65)	938 (73.45)	
	Low active	394 (16.70)	229 (21.16)	165 (12.92)	
	Active	133 (5.64)	82 (7.58)	51 (3.99)	
	highly active	25 (1.06)	19 (1.76)	6 (0.47)	
Physical performance		9.4 ± 1.71	9.97 ± 1.35	8.84 ± 1.84	< 0.0001
Gait Speed (m/sec)		0.84 ± 0.3	1.00 ± 0.26	0.71 ± 0.27	< 0.0001
Handgrip		22.24 ± 9.2	27.62 ± 8.43	17.64 ± 7.11	< 0.0001

*BEH* Bushehr elderly health, *BMI* body mass index, *NC* neck circumferences, *WC* Waist circumferences, *HC* hip circumferences, *WHR* Waist to hip ratio, *LBM* Lean body mass, *ASM* appendicular skeletal muscle, *FM* fat mass, *FFM* fat-free mass, *DS* Dysmobility Syndrome

^a^*P*-values for continuous variables and categorical variables were assessed using t-test and Chi-square, respectively

• *P*-value < 0.05

According to Table 3, the total, trunk, limb, arms, and legs FM and FM to FFM ratio in the DS group were higher than the normal group, while the ratio of trunk to limb FM and android to gynoid FM in DS group were lower than the normal group (*p* < 0.001).

**Table 3:** Body composition components according to having or not having dysmobility syndrome (DS) (*n* = 2,359)

			Normal (*N* = 1,082)	DS (*N* = 1,277)	*p*-value
Limbs	Total	FM%	34.90 ± 9.75	40.88 ± 9.16	< 0.0001
		FM to FFM ratio	0.57 ± 0.26	0.73 ± 0.26	< 0.0001
	Arms	FM%	34.96 ± 11.38	41.65 ± 10.82	< 0.0001
		FM to FFM ratio	0.59 ± 0.32	0.77 ± 0.32	< 0.0001
	Legs	FM%	34.85 ± 9.35	40.53 ± 8.85	< 0.0001
		FM to FFM ratio	0.57 ± 0.25	0.71 ± 0.25	< 0.0001
Trunk	FM%		36.98 ± 7.93	40.56 ± 7.95	< 0.0001
	FM to FFM ratio		0.61 ± 0.20	0.71 ± 0.21	< 0.0001
Total Body	FM%		35.24 ± 7.99	39.54 ± 7.69	< 0.0001
	FM to FFM ratio		0.56 ± 0.20	0.67 ± 0.20	< 0.0001
Trunk to limb FM ratio			1.39 ± 0.27	1.32 ± 0.26	< 0.0001
Android to Gynoid FM ratio			1.16 ± 0.18	1.08 ± 0.16	< 0.0001

*FM* fat mass, *FFM* fat-free mass, *DS* Dysmobility Syndrome

Table 4 represents the association between FM and the FM to FFM ratio in different regions of the body (arms, legs, trunk, limb and total), trunk to limb FM ratio and android to gynoid FM ratio with odds of DS. According to the final logistic regression model in limb region FM and FM to FFM ratio were significantly associated with DS [OR (95%CI) = 1.04 (1.02 to 1.05), *p*-value < 0.001 and OR (95%CI) = 3.42 (1.95 to 5.99), *p*-value < 0.001, respectively]. In the trunk region, the FM and FM to FFM ratio were significantly related to the odds of DS, although this relationship was weaker than limb region [OR (95%CI) = 1.02 (1.00 to 1.03), *p*-value = 0.003 and OR (95%CI) = 2.45 (1.36 to 4.39), *p*-value = 0.003, respectively]. This association was also established in the total body [OR (95%CI) = 1.03 (1.01 to 1.05), *p*-value < 0.001 and OR (95%CI) = 3.56 (1.81 to 6.97), *p*-value < 0.001, respectively].

**Table 4:** Association of body composition components with dysmobility syndrome (DS) (*n* = 2,359)

				*n* = 2,359	
				OR (95% CI)	*p*-value
Limbs	Total	FM (%)	Crude	1.06 (1.05 to 1.07)	< 0.001
			Model 1	1.03 (1.01 to 1.05)	< 0.001
			Model 2	1.03 (1.02 to 1.05)	< 0.001
			Model 3	1.04 (1.02 to 1.05)	< 0.001
			Model 4	1.04 (1.02 to 1.05)	< 0.001
		FM to FFM ratio	Crude	9.43 (6.79 to 13.09)	< 0.001
			Model 1	2.51 (1.48 to 4.23)	0.001
			Model 2	3.13 (1.79 to 5.45)	< 0.001
			Model 3	3.24 (1.86 to 5.66)	< 0.001
			Model 4	3.42 (1.95 to 5.99)	< 0.001
	Arms	FM (%)	Crude	1.05 (1.04 to 1.06)	< 0.001
			Model 1	1.02 (1.00 to 1.03)	0.001
			Model 2	1.02 (1.01 to 1.04)	< 0.001
			Model 3	1.02 (1.01 to 1.04)	< 0.001
			Model 4	1.03 (1.01 to 1.04)	< 0.001
		FM to FFM ratio	Crude	5.57 (4.26 to 7.27)	< 0.001
			Model 1	1.57 (1.03 to 2.39)	0.034
			Model 2	2.01 (1.28 to 3.15)	0.002
			Model 3	2.04 (1.30 to 3.20)	0.002
			Model 4	2.12 (1.35 to 3.34)	0.001
	Legs	FM (%)	Crude	1.06 (1.05 to 1.07)	< 0.001
			Model 1	1.03 (1.02 to 1.05)	< 0.001
			Model 2	1.03 (1.02 to 1.05)	< 0.001
			Model 3	1.03 (1.02 to 1.05)	< 0.001
			Model 4	1.04 (1.02 to 1.05)	< 0.001
		FM to FFM ratio	Crude	10.32 (7.31 to 14.56)	< 0.001
			Model 1	2.67 (1.58 to 4.49)	< 0.001
			Model 2	3.12 (1.80 to 5.40)	< 0.001
			Model 3	3.24 (1.87 to 5.63)	< 0.001
			Model 4	3.40 (1.95 to 5.92)	< 0.001
Trunk	FM (%)		Crude	1.05 (1.04 to 1.06)	< 0.001
			Model 1	1.00 (0.99 to 1.02)	0.299
			Model 2	1.02 (1.00 to 1.03)	0.007
			Model 3	1.02 (1.00 to 1.03)	0.006
			Model 4	1.02 (1.00 to 1.03)	0.003
	FM to FFM ratio		Crude	8.62 (5.79 to 12.82)	< 0.001
			Model 1	1.34 (0.79 to 2.29)	0.273
			Model 2	2.26 (1.27 to 4.03)	0.005
			Model 3	2.29 (1.28 to 4.09)	0.005
			Model 4	2.45 (1.36 to 4.39)	0.003
Total Body	FM (%)		Crude	1.07 (1.05 to 1.08)	< 0.001
			Model 1	1.02 (1.00 to 1.03)	0.012
			Model 2	1.03 (1.01 to 1.05)	< 0.001
			Model 3	1.03 (1.01 to 1.05)	< 0.001
			Model 4	1.03 (1.01 to 1.05)	< 0.001
	FM to FFM ratio		Crude	13.32 (8.80 to 20.16)	< 0.001
			Model 1	1.93 (1.04 to 3.58)	0.036
			Model 2	3.02 (1.64 to 6.23)	0.001
			Model 3	3.29 (1.69 to 6.43)	< 0.001
			Model 4	3.56 (1.81 to 6.97)	< 0.001
Trunk to limb FM ratio			Crude	0.36 (0.26 to 0.49)	< 0.001
			Model 1	0.80 (0.57 to 1.12)	0.211
			Model 2	1.01 (0.70 to 1.45)	0.942
			Model 3	1.00 (0.69 to 1.43)	0.996
			Model 4	1.01 (0.70 to 1.44)	0.952
Android to Gynoid FM ratio			Crude	0.06 (0.04 to 0.11)	< 0.001
			Model 1	0.26 (0.15 to 0.46)	< 0.001
			Model 2	0.43 (0.24 to 0.77)	0.005
			Model 3	0.42 (0.23 to 0.76)	0.004
			Model 4	0.44 (0.24 to 0.79)	0.007

Multivariable logistic regression was used for analysis

Models:

Crude

Model 1 adjusted for sex

Model 2 adjusted for Model 1 + age

Model 3 adjusted for Model 2 + the number chronic diseases ^a^ + income + marital

Model 4 adjusted for Model 3 + smoke + physical activity

*FM* fat mass, *FFM* fat-free mass, *OR* odds ratio, *CI* confidence interval

^a^Chronic disease included: liver diseases, lung diseases, cardiovascular disease, hypertension, diabetes mellitus, thyroid diseases, osteoarthritis, and rheumatoid arthritis, CKD

In addition, in the final models, there was no significant association between trunk to limb FM ratio and DS (*p*-value = 0.952). However, an inverse association between android to gynoid FM ratio and the odds of DS was observed [OR (95%CI) = 0.44 (0.24 to 0.79), *p*-value = 0.007].

**Fig. 1:**
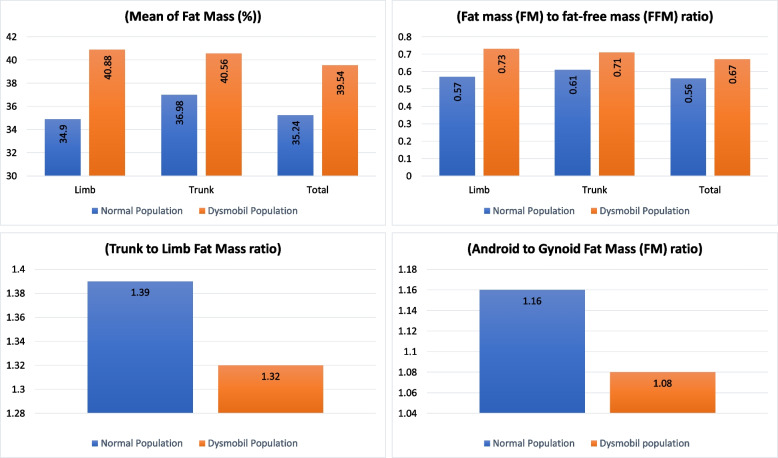
Comparing the Body composition components in the Limb, trunk, and total base on total population. limb, trunk, and total body, mean of fat mass (%); Fat mass (FM) to fat-free mass (FFM) ratio; the trunk to limb fat mass ratio; and Android to gynoid fat mass (FM) ratio in the total population; Bushehr Elderly Health (BEH) program (*n* = 2,359)

Figure 1 shows the differences in FM and the FM to FFM ratio between different regions of the body (trunk, limb and total), trunk to limb FM ratio and android to gynoid FM ratio in DS and normal subjects. In the total population, there were significant differences in the total and regional FM and FM to FFM ratio, between participants with and without DS. Also, significant differences were seen in the trunk to limb and Android to Gynoid FM ratio (*p*-value =  < 0.001).

## Discussion

This cross-sectional study aimed to assess the association between regional FM distribution and incident of DS in an older population. Our results indicated that FM and especially the FM to FFM ratio in different regions of the body (trunk, limbs, arms, legs and total) are associated with increased odds of DS, in such a way that this relationship is stronger for the limbs region. Also, interestingly, a protective association between the android to gynoid FM ratio with the odds of DS was observed.

Interestingly, the average BMI of the DS group in our population was found to be significantly lower than that of the normal group (26.96 vs. 27.81 kg/m2). This observation may be attributed to the fact that although the total amount of FM was similar in both groups, the DS group had significantly lower LBM and ASM, leading to a higher FM% and limiting the efficacy of BMI in accurately reflecting the correlation with DS that highlight the importance of considering the fat distribution indexes instead of BMI. As we know, during the aging process, the occurrence of sarcopenia, or in other words, a decrease in LBM and an increase in body FM, is directly related to the various disabling components of DS [11, 32]. Evidence shows that one of the causes of DS can be increased body fat/obesity [6]. Some studies showed that increase in the android to gynoid ratio FM can be dangerous and increase the risk of fractures, metabolic disorders [33–35], lower BMD [36–38], sarcopenia and reduced physical performance [39]. In the case of osteoporosis, the evidence is somewhat contradictory and shows that body weight, including fat and lean mass, contribute to higher bone density [40], although meta-analysis studies indicate a greater effect of lean mass in this regard [41].

Based on our results, the numbers of individuals with lower physical activity tend to be higher in those diagnosed with DS. With the passage of time and the natural aging process, the human body experiences a series of changes that have implications for both muscle mass and functionality. Physical activity has the potential to mitigate the adverse impacts of aging on muscle mass and function. Research indicates that both aerobic and strength training can significantly enhance strength and motor performance in older individuals [42–44]. A significant portion of the aging population worldwide, similar to the findings of our study, encounter numerous obstacles that impede their capacity to participate in sufficient levels of physical activity, thereby limiting their physical engagement [45]. As individuals age beyond 40 years, they may experience declining physiological function, accompanied by anatomical and ultrastructural changes. These changes can manifest as cognitive decline affecting memory and learning, skeletal muscle atrophy causing progressive weakness (sarcopenia), and reduced bone mineral density leading to osteopenia and osteoporosis [46].

The primary objective of our research study was to investigate the potential impact of the distribution of FM throughout different regions of the body on the odds of developing DS. In a recent study [47], it was found that abdominal visceral fat has an inverse relationship with bone density, and in this regard, increasing insulin resistance has been proposed as a mechanism. However, limbs FM was not measured in that study and the participants were non-older adults. In the present study, we observed for the first time that in older people, more FM in the limbs than in the trunk region is associated with greater odds of DS, and in line with that, a greater ratio of gynoid to android fat increases this odd. On the other hand, it was seen that trunk to limb and android to gynoid FM ratio was lower in DS than in normal population. Furthermore, our data indicates that the buildup of FM relative to FFM in both the upper and lower limbs is linked to DS. Interestingly, although the correlation between the lower limbs and DS is more robust, the upper limbs are also strongly associated with DS, with respective odds ratios of 2.12 and 3.40. We speculated that these new findings can be attributed to increased limbs intramuscular FM in older people. Intramuscular fat accumulation is considered as a predictor of decline in muscle function and motor ability in older people [48]. Intramuscular fat, similar to visceral fat, has the ability to release pro-inflammatory cytokines such as interleukin-6, which leads to muscular inflammation and decrease in muscle and mobility function [49]. Also, the accumulation of limbs intramuscular fat leads to an increase in insulin resistance through a decrease in muscle blood flow, an increase in the rate of lipolysis and glucose accumulation, and alteration in mitochondrial action [50, 51]. Consistent with our results, a study by Neri et al., which was conducted on Brazilian older women, revealed that gynoid FM was associated with an increased risk of falls in women over 60 years of age [52] and also in another study it was found that participants with gynoid obesity had lower knee extensors peak torque [53]. Given these findings from previous studies, it does not seem unreasonable that limbs and gynoid FM is more related to various functional and bone disorders of DS in old age. The results of the present study support the importance of limbs and gynoid FM accumulation in the incident DS.

Regarding the adjusted variables in the statistical models, age and sex as background variables based on previous studies were associated with DS [19, 54, 55]. Also chronic disease contribute to DS and mortality and were adjusted in model 3 [15]. After that, smoking and physical activity were included in the model, which showed that these factors can affect health or lifestyle in the DS population [15, 19].

To the best of our knowledge, this study was the first to examine the association between body fat distributions and DS, with data derived from a large-scale population-based cohort study. The prevalence of the DS in our study population was estimated as approximately 54%, which was higher than previous studies (22%–34%) [11]. The high prevalence of DS in this Iranian elderly community shows the importance of conducting such studies and implementing relevant interventions to address contributory factors. Also in these subjects we found a higher risk of osteosarcopenia, risk of falls and fractures [56]. This study examined a large sample of older people in Iran using an established protocol, including that we evaluated both measured body composition indices of different anthropometric indices and DXA with standard methods. Nevertheless, we were faced with some limitations, including the cross-sectional nature of the study which cannot show the cause and effect relationship, and the effect of unmeasured confounders such as drug usage and nutritional status of participants.

## Conclusion

Higher body FM is associated with a higher risk of the DS. Also, interestingly, our results show that in older people, higher limbs FM is associated with a greater odd of DS, in a way that a higher ratio of android to gynoid FM is associated with a lower risk of the DS. Therefore, the screening of FM distribution in older people can be a valuable complementary strategy for the rapid diagnosis of the DS and introduction of interventions for the prevention of related disabilities and improving the quality of life in this population.

## Data Availability

The corresponding Author or IN (inabipour@gmail.com), upon reasonable request, has the datasets used for the current study available.

## Abbreviations

BEH: Bushehr elderly health
AHA: American Heart Association
SBP: Systolic blood pressure
DBP: Diastolic blood pressure
BMI: Body mass index
WHR: Waist to hip ratio
HTN: Hypertension
HDL: High-density lipoproteins
LDL: Low-density lipoproteins
TG: Triglycerides
TC: Total cholesterol
NC: Neck circumferences
WC: Waist circumferences
HC: Hip circumferences
WHR: Waist to hip ratio
FM: Fat mass
FFM: Fat-free mass
LBM: Lean body mass
ASM: Appendicular skeletal muscle
DXA: Dual-energy X-ray absorptiometry

## References

[1] Santos AL, Sinha S (2021). Obesity and aging: molecular mechanisms and therapeutic approaches. Ageing Res Rev.

[2] Salvestrini V, Sell C, Lorenzini A (2019). Obesity may accelerate the aging process. Front Endocrinol.

[3] Hruby A, Hu FB (2015). The epidemiology of obesity: a big picture. Pharmacoeconomics.

[4] Rossi-Izquierdo M, Santos-Pérez S, Faraldo-García A, Vaamonde-Sánchez-Andrade I, Gayoso-Diz P, Del-Río-Valeiras M (2016). Impact of obesity in elderly patients with postural instability. Aging Clin Exp Res.

[5] Vincent HK, Vincent KR, Lamb KM (2010). Obesity and mobility disability in the older adult. Obes Rev.

[6] Binkley N, Krueger D, Buehring B (2013). What’s in a name revisited: should osteoporosis and sarcopenia be considered components of “dysmobility syndrome?”. Osteoporos Int.

[7] Hill KD, Farrier K, Russell M, Burton E (2017). Dysmobility syndrome: current perspectives. Clin Interv Aging.

[8] Lee W-J, Liu L-K, Hwang A-C, Peng L-N, Lin M-H, Chen L-K (2017). Dysmobility Syndrome and Risk of Mortality for Community-Dwelling Middle-Aged and Older Adults: The Nexus of Aging and Body Composition. Sci Rep.

[9] Lee W-J, Liu L-K, Hwang A-C, Peng L-N, Lin M-H, Chen L-K (2017). Dysmobility syndrome and risk of mortality for community-dwelling middle-aged and older adults: the nexus of aging and body composition. Sci Rep.

[10] Lim E, Noh J (2015). Physical function, cognitive function, and depressive symptoms in elderly women with dysmobility syndrome. Int J Bio-Science Bio-Technology.

[11] Hill KD, Farrier K, Russell M, Burton E (2017). Dysmobility syndrome: current perspectives. Clin Interv Aging.

[12] Burgueno-Aguilar K, Cons-Molina FF, Garcia-Jimenez D, Bejarano-Lopez LE, Gudino-Barroso MA (2021). Dysmobility syndrome: a case-series study describing a musculoskeletal syndrome in postmenopausal Mexican women. Arch Osteoporos.

[13] Fahimfar N, Zahedi Tajrishi F, Gharibzadeh S, Shafiee G, Tanha K, Heshmat R (2020). Prevalence of osteosarcopenia and its association with cardiovascular risk factors in Iranian older people: Bushehr Elderly Health (BEH) Program. Calcif Tissue Int.

[14] Clynes M, Edwards M, Buehring B, Dennison E, Binkley N, Cooper C (2015). Definitions of sarcopenia: associations with previous falls and fracture in a population sample. Calcif Tissue Int.

[15] Looker AC (2015). Dysmobility syndrome and mortality risk in US men and women age 50 years and older. Osteoporos Int.

[16] Iolascon G, Moretti A, Giamattei MT, Migliaccio S, Gimigliano F (2015). Prevalent fragility fractures as risk factor for skeletal muscle function deficit and dysmobility syndrome in post-menopausal women. Aging Clin Exp Res.

[17] Lee W-J, Peng L-N, Chiou S-T, Chen L-K (2016). Relative handgrip strength is a simple indicator of cardiometabolic risk among middle-aged and older people: a nationwide population-based study in Taiwan. PLoS ONE.

[18] Chen Y-Y, Kao T-W, Wang C-C, Chen Y-J, Wu C-J, Chen W-L (2018). Exploring the link between metabolic syndrome and risk of dysmobility syndrome in elderly population. PLoS ONE.

[19] Dos Santos VR, Diniz TA, Batista VC, Júnior IFF, Gobbo LA (2019). Practice of physical activity and dysmobility syndrome in community-dwelling older adults. J Exercise Rehabil.

[20] Lee W-J, Liu L-K, Peng L-N, Lin M-H, Chen L-K, Group IR (2013). Comparisons of sarcopenia defined by IWGS and EWGSOP criteria among older people: results from the I-Lan longitudinal aging study. J Am Med Directors Assoc..

[21] Mitchell RJ, Lord SR, Harvey LA, Close JC (2014). Associations between obesity and overweight and fall risk, health status and quality of life in older people. Aust N Z J Public Health.

[22] Colleluori G, Villareal DT (2021). Aging, obesity, sarcopenia and the effect of diet and exercise intervention. Exp Gerontol.

[23] Gkastaris K, Goulis DG, Potoupnis M, Anastasilakis AD, Kapetanos G (2020). Obesity, osteoporosis and bone metabolism. J Musculoskelet Neuronal Interact.

[24] Landi F, Liperoti R, Russo A, Giovannini S, Tosato M, Capoluongo E (2012). Sarcopenia as a risk factor for falls in elderly individuals: results from the ilSIRENTE study. Clin Nutr.

[25] Chen H, Yi Y-Y, Zhang S-B, Xu H-W, Fang X-Y, Tao H (2022). Sarcopenic obesity defined by visceral adiposity was associated with osteoporotic vertebral fracture. Arch Osteoporos.

[26] Namwongprom S, Rojanasthien S, Wongboontan C, Mangklabruks A (2019). Contribution of Android and Gynoid Adiposity to Bone Mineral Density in Healthy Postmenopausal Thai Women. J Clin Densitom.

[27] Cheng S-H, Kuo Y-J, Lin JC-F, Chang W-C, Wu C-C, Chu Y-L (2020). Fat distribution may predict intra- or extra-capsular hip fracture in geriatric patients after falling. Injury..

[28] Ostovar A, Nabipour I, Larijani B, Heshmat R, Darabi H, Vahdat K (2015). Bushehr elderly health (BEH) Programme, phase I (cardiovascular system). BMJ Open.

[29] Shafiee G, Ostovar A, Heshmat R, Darabi H, Sharifi F, Raeisi A (2017). Bushehr Elderly Health (BEH) programme: study protocol and design of musculoskeletal system and cognitive function (stage II). BMJ Open.

[30] Kelishadi R, Rabiei K, Khosravi A, Famouri F, Sadeghi M, Rouhafza H, et al. Assessment of physical activity of adolescents in Isfahan. J Shahrekord Univ Med Sci. 2001;3(2):27–33. http://78.39.35.44/article-1-685-en.html.

[31] Aadahl M, Jørgensen T (2003). Validation of a new self-report instrument for measuring physical activity. Med Sci Sports Exerc.

[32] Lee W-J, Liu L-K, Hwang A-C, Peng L-N, Lin M-H, Chen L-K. Dysmobility Syndrome and Risk of Mortality for Community-Dwelling Middle-Aged and Older Adults: The Nexus of Aging and Body Composition. Sci Rep. 2017;7(1):8785. 10.1038/s41598-017-09366-z. 10.1038/s41598-017-09366-z PMC5562709 28821868

[33] Zheng R, Byberg L, Larsson SC, Höijer J, Baron JA, Michaëlsson K (2021). Prior loss of body mass index, low body mass index, and central obesity independently contribute to higher rates of fractures in elderly women and men. J Bone Miner Res.

[34] Tsutsumimoto K, Makizako H, Hotta R, Nakakubo S, Makino K, Suzuki T (2018). Cognitive frailty is associated with fall-related fracture among older people. J Nutr Health Aging.

[35] Vasan SK, Osmond C, Canoy D, Christodoulides C, Neville MJ, Di Gravio C (2018). Comparison of regional fat measurements by dual-energy X-ray absorptiometry and conventional anthropometry and their association with markers of diabetes and cardiovascular disease risk. Int J Obes.

[36] Tchernof A, Desmeules A, Richard C, Laberge P, Daris M, Mailloux J (2004). Ovarian hormone status and abdominal visceral adipose tissue metabolism. J Clin Endocrinol Metab.

[37] Xiao Z, Tan Z, Shang J, Cheng Y, Tang Y, Guo B (2020). Sex-specific and age-specific characteristics of body composition and its effect on bone mineral density in adults in southern China: a cross-sectional study. BMJ Open.

[38] Xiao Z, Xu H. Gender-Specific Body Composition Relationships between Adipose Tissue Distribution and Peak Bone Mineral Density in Young Chinese Adults. BioMed Research International. 2020;2020:6724749. 10.1155/2020/6724749. 10.1155/2020/6724749 PMC7152964 32337266

[39] Abbas H, Perna S, Shah A, Al-Mannai M, Gasparri C, Infantino V (2020). Risk factors for 5-year mortality in a cohort of elderly patients with sarcopenia. Exp Gerontol.

[40] Ho-Pham LT, Nguyen ND, Lai TQ, Nguyen TV (2010). Contributions of lean mass and fat mass to bone mineral density: a study in postmenopausal women. BMC Musculoskelet Disord.

[41] Ho-Pham LT, Nguyen UDT, Nguyen TV (2014). Association Between Lean Mass, Fat Mass, and Bone Mineral Density: A Meta-analysis. J Clin Endocrinol Metab.

[42] Chen S, Ling J, Cheng Y (2023). Physical activity and body mass index were interactively related to health-related quality of life among older adults. Arch Gerontol Geriatr.

[43] Cvecka J, Tirpakova V, Sedliak M, Kern H, Mayr W, Hamar D (2015). Physical Activity in Elderly. Eur J Transl Myol.

[44] Moreno-Agostino D, Daskalopoulou C, Wu Y-T, Koukounari A, Haro JM, Tyrovolas S (2020). The impact of physical activity on healthy ageing trajectories: evidence from eight cohort studies. Int J Behav Nutr Phys Act.

[45] Milanović Z, Pantelić S, Trajković N, Sporiš G, Kostić R, James N (2013). Age-related decrease in physical activity and functional fitness among elderly men and women. Clin Interv Aging.

[46] McPhee JS, French DP, Jackson D, Nazroo J, Pendleton N, Degens H (2016). Physical activity in older age: perspectives for healthy ageing and frailty. Biogerontology.

[47] Hilton C, Vasan SK, Neville MJ, Christodoulides C, Karpe F (2022). The associations between body fat distribution and bone mineral density in the Oxford Biobank: a cross sectional study. Expert Rev Endocrinol Metab.

[48] Addison O, Marcus RL, LaStayo PC, Ryan AS (2014). Intermuscular Fat: A Review of the Consequences and Causes. Int J Endocrinol.

[49] Addison O, Drummond MJ, LaStayo PC, Dibble LE, Wende AR, McClain DA (2014). Intramuscular fat and inflammation differ in older adults: the impact of frailty and inactivity. J Nutr Health Aging.

[50] Therkelsen KE, Pedley A, Speliotes EK, Massaro JM, Murabito J, Hoffmann U (2013). Intramuscular Fat and Associations With Metabolic Risk Factors in the Framingham Heart Study. Arterioscler Thromb Vasc Biol.

[51] Yim J, Heshka S, Albu J, Heymsfield S, Kuznia P, Harris T (2007). Intermuscular adipose tissue rivals visceral adipose tissue in independent associations with cardiovascular risk. Int J Obes.

[52] Neri SG, Tiedemann A, Gadelha AB, Lima RM (2020). Body fat distribution in obesity and the association with falls: a cohort study of Brazilian women aged 60 years and over. Maturitas.

[53] Neri SG, Pereira JC, de David AC, Lima RM (2021). The influence of body fat distribution on postural balance and muscle quality in women aged 60 years and over. J Appl Biomech.

[54] Jung Y, Hong N, Kim C, Kim H, Youm Y, Choi J-Y (2021). The diagnostic value of phase angle, an integrative bioelectrical marker, for identifying individuals with dysmobility syndrome: The Korean Urban-Rural Elderly study. Osteoporos Int.

[55] Chen F-P, Lin-Jr Y, Chao A-S, Lin Y-C, Sung C-M, Chen J-F, et al. Utilizing nomograms to predict prevalent vertebral fracture risk: an analysis of dysmobility syndrome in a community-dwelling population. Biomed J. 2022;45(6):931–9. 10.1016/j.bj.2021.11.008. 10.1016/j.bj.2021.11.008 PMC9795362 34801764

[56] Fathi M, Heshmat R, Ebrahimi M, Salimzadeh A, Ostovar A, Fathi A (2021). Association between biomarkers of bone health and osteosarcopenia among Iranian older people: The Bushehr Elderly Health (BEH) program. BMC Geriatr.

